# Short Term Evolution of a Highly Transmissible Methicillin-Resistant *Staphylococcus aureus* Clone (ST228) in a Tertiary Care Hospital

**DOI:** 10.1371/journal.pone.0038969

**Published:** 2012-06-18

**Authors:** Valérie Vogel, Laurent Falquet, Sandra P. Calderon-Copete, Patrick Basset, Dominique S. Blanc

**Affiliations:** 1 Service of Hospital Preventive Medicine, Lausanne University Hospital, Lausanne, Switzerland; 2 Vital-IT Group, Swiss Institute of Bioinformatics, Lausanne, Switzerland; University of Florida, United States of America

## Abstract

*Staphylococcus aureus* is recognized as one of the major human pathogens and is by far one of the most common nosocomial organisms. The genetic basis for the emergence of highly epidemic strains remains mysterious. Studying the microevolution of the different clones of *S. aureus* is essential for identifying the forces driving pathogen emergence and spread. The aim of the present study was to determine the genetic changes characterizing a lineage belonging to the South German clone (ST228) that spread over ten years in a tertiary care hospital in Switzerland. For this reason, we compared the whole genome of eight isolates recovered between 2001 and 2008 at the Lausanne hospital. The genetic comparison of these isolates revealed that their genomes are extremely closely related. Yet, a few more important genetic changes, such as the replacement of a plasmid, the loss of large fragments of DNA, or the insertion of transposases, were observed. These transfers of mobile genetic elements shaped the evolution of the ST228 lineage that spread within the Lausanne hospital. Nevertheless, although the strains analyzed differed in their dynamics, we have not been able to link a particular genetic element with spreading success. Finally, the present study showed that new sequencing technologies improve considerably the quality and quantity of information obtained for a single strain; but this information is still difficult to interpret and important investments are required for the technology to become accessible for routine investigations.

## Introduction

*Staphylococcus aureus* is recognized as one of the major human pathogen and is by far one of the most common nosocomial organisms. This bacterium can cause a variety of infections ranging from superficial skin infections to severe life-threatening conditions such as bacteraemia [1], endocarditis, pneumonia and toxic shock syndrome. *S. aureus* has amazing capacities to survive and adapt in hostile environments, as illustrated by the emergence of *S. aureus* strains resistant to almost all classes of antimicrobials [2]. In particular, methicillin-resistant *Staphylococcus aureus* (MRSA) represents a major issue both in health care settings (HA-MRSA) and in the community (CA-MRSA), and numerous MRSA outbreaks have been reported worldwide. Epidemiological studies revealed that MRSA infections were mostly caused by single clones that spread rapidly in the hospital settings or recently in the community (epidemic MRSA). The reason why a single MRSA clone can be common and widespread in a large geographic area while other clones are restricted to few sporadic isolates remains mysterious. However, the dissemination of particular clones in a specific environment in favor of other strains [3]–[5] or the replacement of clones in a single environment [6], [7] suggests a genetic basis for epidemicity. Thus, the speed at which genetic changes accumulate as well as the frequency at which entire genes, or complex mobile genetic elements are gained and lost are crucial parameters for understanding the mechanisms by which epidemicity, pathogenicity, virulence, or antibiotic resistance might be acquired.

The *S. aureus* population consists of about ten major lineages associated to humans and many other minor lineages [8]. Genome comparisons performed so far indicated that isolates from different lineages differ at hundreds of genes [9]–[11] while isolates from the same lineage have remarkably conserved genomes despite wide geographic, temporal and presumably selective diversity [12]–[14]. For example, only 285 points mutations among approximately 2500 core genes distinguish two *S. aureus* genomes belonging to the same lineage [12]. Similarly, comparative whole genome sequencing of ten USA300 isolates collected nationwide in the USA showed a limited number of single nucleotide polymorphisms and regions of differences among these isolates [15]. More recently, Harris *et al.* [16] used high-throughput sequencing to compare the genome of 63 isolates of a predominant clonal lineage (ST239) common in mainland Asia, South America and parts of Eastern Europe. They also found a relatively low number of variable sites (around 4300) in the core genome and this variation was sufficient to infer the evolutionary history of the lineage. A complementary analysis of these data using Markov spatial models demonstrated that bacterial genomes could indeed contain sufficient evolutionary information to elucidate the temporal and spatial dynamics of transmission of this lineage [17]. These studies did not only provide new clues about the geographical origin, international spread and local epidemiology of this clonal lineage; it also highlighted the potential of whole-genome sequencing technologies to infer the local epidemiology and short term evolution of pathogens.

**Figure 1:**
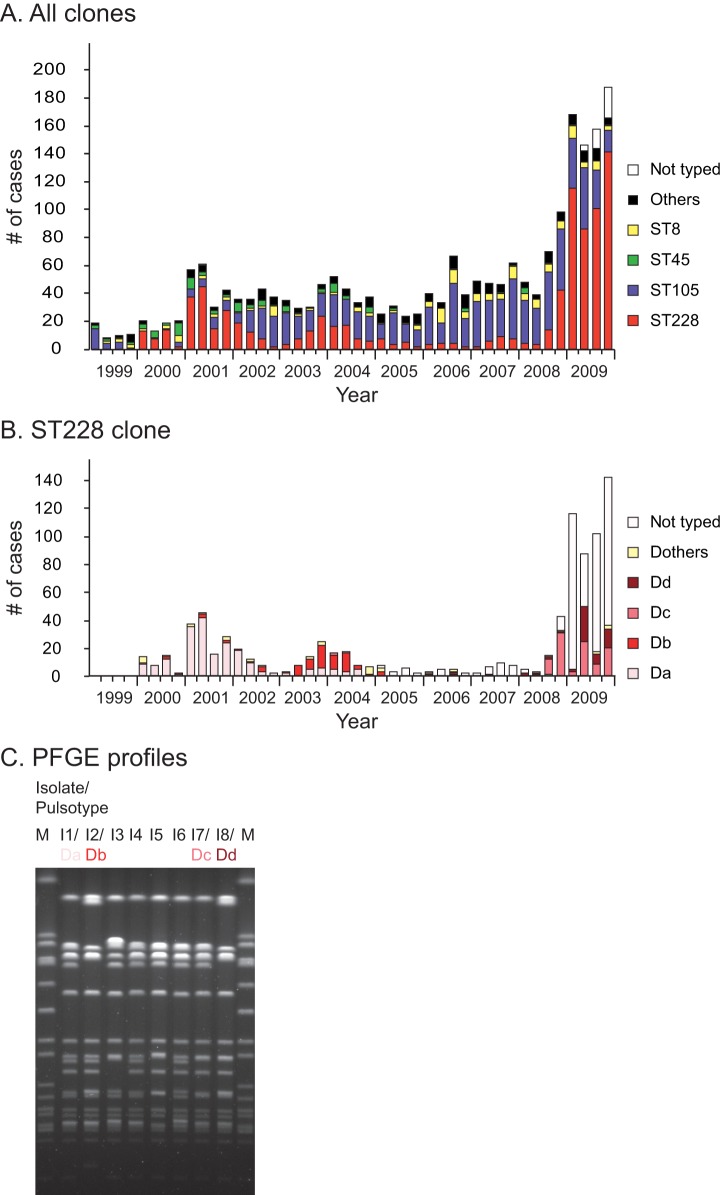
MRSA survey at the tertiary care hospital of Lausanne. Molecular typing was performed by Pulsed Field Gel Electrophoresis (PFGE) until 2005 and then by Double Locus Sequence Typing (DLST; [51]). A. Trimestral incidence of patients with MRSA between 1999 and 2009. The four main lineages of MRSA encountered at the hospital are labeled by colors; the ST228 in red, the ST105 in violet, the ST45 in green and the ST8 in yellow. The other lineages, less common at the hospital, are in black. B. Trimestral incidence of patients with MRSA of clone ST228 at the University hospital of Lausanne between 1999 and 2009. A subset of MRSA detected between 2008 and 2009 were randomly selected for PFGE typing. The four most common pulsotypes (Da, Db,Dc and Dd) were labeled in red-like colors. C. PFGE profile of the eight isolates selected for the genome comparison. Among these eight isolates, we selected the four most common pulsotypes described in B. The eight pulsotypes were bordered by a marker (M) corresponding to the restriction of the reference strain NCTC 8325.

The ST228-MRSA-I, also called the South German clone or Italian clone [18], is prevalent in several central European countries including Germany [19], Italy [18], Hungary [20], Slovenia [21], Austria [21] and Switzerland [7]. In Switzerland, it was first detected at the end of the 90’s [7]. Since then, it was regularly observed at the Geneva and Lausanne tertiary care hospitals but rarely found in other places in Switzerland. Interestingly, the importance of this clone varies not only geographically but also temporarily. Short after its arrival at the tertiary care hospital of Lausanne, the ST228 clone was responsible for two important outbreaks (Fig. 1B). Four years later, the prevalence of this clone diminishes drastically and it became rare compared to other lineages. Variants of this clone were regularly observed but never spread such extensively until 2008. During the last trimester of 2008, the unusual dissemination of a strain belonging to ST228 was observed. This strain affected more than five hundred patients in less than one year and a half, whereas other clones did not disseminate as extensively although they were in the same setting. The surveillance program established at the Lausanne hospital permitted to follow the dynamic of this clone over ten years providing the ideal material for studying the local microevolution of an epidemic MRSA clone (Fig. 1).

The aim of the present study was to determine the genetic changes characterizing the ST228 lineage that spread over ten years in a single hospital in order to understand its short-term evolution as well as its local dynamic. To determine the genetic changes that occurred during the spread of this lineage, we compared the whole genome of eight isolates recovered at the Lausanne tertiary care hospital between 2001 and 2008. In addition, we used these data to test the utility of next generation sequencing for very localized epidemiology. The comparison of these eight genomes revealed that they evolved mainly through point mutations accompanied by few transfers of mobile genetic elements (MGEs). None of these changes was characteristic of the most successful isolates suggesting that there is probably not a single genetic factor explaining the particular spreading success of some of the ST228 variants found at the hospital. Finally, the present study confirmed that whole genome comparison improves considerably the genetic characterization of a lineage at a very local epidemiological level but requires a broader knowledge on the evolution of the linage to draw reliable interpretation of the results for epidemiological tracking.

## Materials and Methods

### Selection of Isolates for Genome Sequencing

Eight different isolates of *Staphylococcus aureus* belonging to ST228 (*spa* type t041) collected between 2001 and 2008 at the tertiary care hospital of Lausanne were selected for genomes comparison (Table 1). During this period, about 550 ST228 isolates were identified at the Lausanne hospital (Fig. 1A). Pulsed-field gel electrophoresis (PFGE) typing of a subset of these 550 isolates (n = 320) identified four predominant PFGE profiles, with an addition of about 20 non-predominant profiles (Fig. 1B). The eight isolates were chosen among these 550 isolates in order to (i) represent the best overview of the evolutionary history of the clone by selecting isolates over the eight years period, (ii) account for the greatest genetic variability by choosing strains having different PFGE profiles, and finally (iii) we selected isolates presenting various dynamics to check if there are genetic changes associated with the predominant variants. According to these criterions, we selected one isolate of each of the four major pulsotypes (Fig. 1B) plus four other isolates that have been observed only sporadically. For each of the predominant pulsotypes, we selected one isolate among those that were first recovered at the hospital during the outbreaks; two isolates were collected in 2001 (Da and Db respectively; Fig. 1B and 1C) and two in 2008 (Dc and Dd; Fig. 1B and 1C). The remaining four sporadic isolates were selected during the non-outbreak period; three isolates were chosen in 2006 and one in 2008 just before the beginning of the outbreak of 2008 (Table 1).

**Table 1:** Information on the eight isolates selected for the whole genome sequencing.

Isolate		Accession number		
Name	Year	Strain #	Genome	Plasmid
**I1**	2001	H10388	HE579059	HE579060
**I2**	2001	H10497	HE579061	HE579062
**I3**	2006	H15532	HE579063	HE579064
**I4**	2006	H16035	HE579065	HE579066
**I5**	2006	H16125	HE579067	HE579068
**I6**	2008	H18341	HE579069	HE579070
**I7**	2008	H18412	HE579071	HE579072
**I8**	2008	H18583	HE579073	HE579074

Name refers to the name of the isolates used in the present paper; Year corresponds to the year of isolation. References concerning these isolates are given as follow: Strain # corresponds to the isolate number in the collection of the tertiary care hospital of Lausanne, EBI genome and plasmid correspond to the accession numbers of the sequences of the main alignment and the plasmid deposited at ENA.

### Genome Sequencing

Total genomic DNA was isolated from the eight isolates (Table 1) using the phenol chloroform extraction method. Genome sequencing was performed with the Illumina sequencing technology (Genome Analyzer IIx) at the Genomic Technologies Facility of the University of Lausanne. A paired-end library with approximately 600 bp insert was constructed from 5 µg of genomic DNA and 36 bp were obtained on both ends following manufacturer’s instructions (Illumina, San Diego, CA). Each isolate was run on a single lane generating from five to eight million reads. In these conditions, the theoretical coverage based on the published genome size of *S. aureus* (*c. a.* 2.8×10^6^) varied from 128× to 205×.

### Assembly

The quality of the data obtained from the sequencing was verified using FastQC [http://www.bioinformatics.bbsrc.ac.uk/projects/fastqc/]. Most of the reads were of excellent quality; reads of insufficient quality or contaminant sequences (less than 1%) were removed using locally developed scripts (available upon request).

To have an overview of the genome of the eight isolates, we preformed a first assembly by mapping the reads of each isolate against a reference for which the genome was known using Bowtie [22]. The strain N315 was used as reference for the genome assembly because it was the most closely related strain for which the whole genome was known [23]. These assemblies revealed that a few elements of the strain N315 were missing in the eight isolates and that, the eight isolates harbored elements not existing in N315. To improve the quality of the assembly, to identify other differences such as genome rearrangement and to determine the nature of the non-mapped elements, we developed a new procedure combining different approaches.

To reconstruct the genomes of our eight isolates, we used a combination of *de novo* assembly (*i.e.*, assembling reads together so that they form a new, previously unknown sequence) and a mapping approach (*i.e*, assembling reads or contigs against a reference). The strain N315 was again used as reference for the genome assembly [23]. First, the reads of one isolate (I1_2001_) were assembled with SOAPdenovo [24]. Then, the resulting contigs were mapped to the genome sequence of the strain N315 (accession number BA000018) with the Smith-Waterman algorithm implemented in BWA-SW [25]. Finally, the gaps were closed by combining various assemblies manually in Consed [26]. With this approach, we constructed a first genome that was further used as reference to assemble the genomes of the seven other isolates using the same method. All genomes were corrected with PrInSeS-G [27], a software tool that detects and assembles sequence variants (1 base pair (bp) to ∼10 kilobases (kb)) from single- or paired-end short reads. The eight assembled genomes were annotated using an internally developed pipeline [28] and compared to each other in order to identify the elements that differ among our eight isolates. From this procedure, we identified the elements that were common to the sequenced and reference strains and those elements that were present in the reference but absent in our isolates. The reads that did not map onto the reference were assembled separately by a *de novo* approach using SOAPdenovo [24]. The resulting contigs constituted the elements that were present in our isolates but not in the reference. The sequences of the isolates I1_2001_ to I8_2008_ resulting from this assembly were deposited at the European Nucleotide Archive (ENA); the accession numbers of each isolate are given in Table 1.

### Phylogeny

To infer the genetic relationships among our eight isolates we performed a phylogenetic analysis. To avoid that horizontal transfer of MGEs confound phylogenetic interpretations; we based our phylogenies on the SNPs located on the core genome defined as the elements present in the eight isolates and in the reference strain N315. We considered that these elements were likely acquired by a common ancestor while those that are missing in one or several isolates might be MGEs lost or acquired during the evolution of the selected strains. The strain N315, which was the most closely related strain for which the whole genome was known [23], was included in the analysis and was used as outgroup for rooting the phylogenetic tree. This analysis was exclusively based on a multiple sequence alignment of the core genome (see below); thus small indels (<200 bp) and the regions varying between the two lineages (SCC*mec*, Φ N315, SaPIn1/SaPlm1, plasmids and ST228 phages; see results section for more details) were excluded from the analysis by a masking procedure. The assembled genomes were first aligned using MUGSY [29] and then the regions of the multiple sequence alignment showing indels (insertions or deletions) were masked with “N”. At this stage the column differences (SNPs) were identified. The phylogenetic tree of this “core genome” was then constructed using RAxML using an approximation of the GTR model with a gamma correction for among site rate variations (GTRCAT) [30]. In this software, support for nodes was assessed by bootstrapping with the autoMRE option of automatic “bootstrapping” using majority-rule tree based criteria [31].

**Figure 2:**
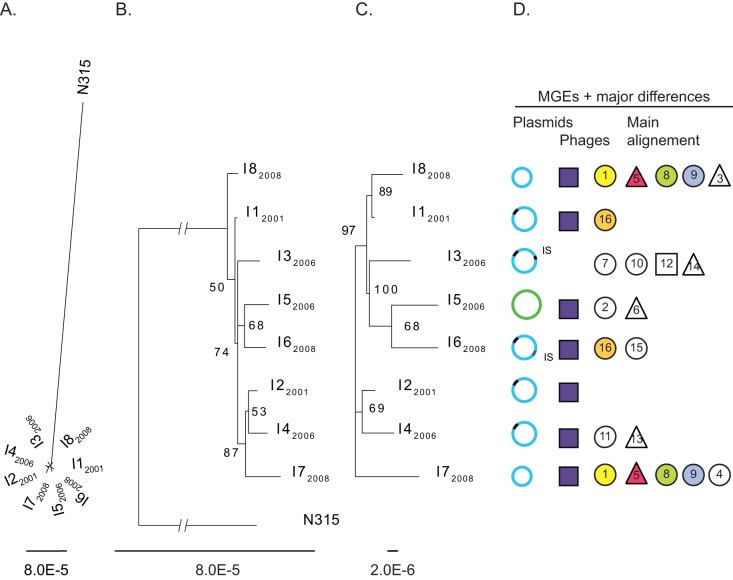
RaxML phylogeny based on the SNPs obtained after the alignment of the core genome. A. In this analysis, the strain N315 was included and used as outgroup. B. Same analysis as in A with bootstrap values given for each node. Nodes that were under the 50% majority rule were collapsed. The tree was rooted with N315 but the distance between this strain and the other was shortened to focus on the relationships between the eight isolates. C. Phylogeny based on the SNPs of the core genome based on the comparison of the eight isolates. D. Mapping of the MGEs and major differences observed between the different isolates onto the phylogenetic tree. Plasmids are represented by circle. The light blue and green circles represent the plasmids that are closely related to the published plasmid SAP064A and pUSA03, respectively. The black trait at the left side of the plasmids represents the presence of the mercuric operon whereas those on the right represent insertions of IS elements. The purple square illustrates the presence of an element of phage of about 18’500 bp. The smaller symbols describe the genetic variations described in Table 2. Circles were used for deletions, triangles for insertions and squares for other types of modifications. The numbers within each symbol correspond to the genetic variation described in Table 2. The symbols are white when the difference is unique to one isolate and colored when it is shared between different isolates.

The resolution of the tree obtained was not sufficient to be confident about the genetic relationship among the eight isolates (Fig. 2A and 2B). In this analysis, large regions were removed from the alignment (*e.g.*, the SCC*mec* region) because they differed between our isolates and N315. The exclusion of these elements reduced the number of informative differences which may have consequences on the resolution of the tree. To assess the impact of this last point, we also analyzed the eight isolates without N315 using the same approach as described above. Finally, to see if large-scale genomic changes followed the evolutionary history of the ST228 lineage, we mapped the MGEs (*i.e.*, elements not present in the eight isolates) and the major differences (*i.e.*, differences observed between the eight isolates that affected more than three base pairs, see Table 2) on the phylogenies.

**Table 2:** Major differences observed between the eight isolates.

				Isolate										
	Position			I1	I2	I3	I4	I5	I6	I7	I8	Type of difference		Localization
**1**	36888		37016							**x**	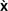	deletion of 120 bp	NC	in SCC*mec* between SACOL0030 and SACOL0031
**2**	242157		242238					**x**				deletion of 82 bp	C	in *coa*
**3**	320106		320180								**x**	insertion of 2×22 bp	C	in pathogenicity island SAPIn2 (SA0289)
**4**	528194		528198							**x**		deletion of 5 bp	NC	between SArRNA05 and SArRNA06
**5**	528467		528560							**x**	**x**	insertion of 93 bp	NC	between SArRNA05 and SArRNA06
**6**	528704		528753					**x**				insertion of 44 bp	NC	between SArRNA05 and SArRNA06
**7**	583658		583695			**x**						deletion of 37 bp	C	in *sdrC*
**8**	647999		648104							**x**	**x**	deletion of 105 bp	C	hypothetical protein similar to NADH deshydrogenase (SA0578)
**9**	804975		805076							**x**	**x**	deletion of 100 bp	NC	between SA0722 and *clpP*
**10**	1049313		1049321			**x**						deletion of 9 bp	C	hypothetical protein (SA0943)
**11**	1116079		1116136				**x**					deletion of 57 bp	NC	between SA1004 and SAS035
**12**	1505038		1506302			**x**						important differencesover 1236 bp	C	hypothetical protein (SA1320)
**13**	2011230		2011626				**x**					insertion of 189 bp	C	hypothetical phage protein (SA1781)
**14**	2340060		2341220			**x**						insertion of 61 bp	C	similar to transcription regulator LysR family (SA2123)
**15**	2569818		2569833						**x**			deletion of 14 bp	C	in *feoB*
**16**	2759629		2759646	**x**					**x**			deletion of 18 bp	NC	between *thdF* and *rnpA*

A difference is considered as major when it affected more than three base pairs. The position of the differences is given relative to the alignment of the eight isolates. The presence of the difference is indicated for each isolate by a cross. The type of difference (deletions, insertions or other) is described. If the difference occurred in a non-coding region it was labeled by a NC and by C when it occurred in a coding region. Finally the region or gene affected by the variation is given.

To understand why the resolution of the trees obtained was low (Fig. 2A, 2B and 2C), we performed two additional analyses. First, we quantified the phylogenetic signal of the two data sets (*i. e.,* core genomes with and without N315) by using the likelihood mapping approach implemented in TREE-PUZZLE [32]. This method displays phylogenetic information contained in a sequence alignment by representing the tree-likeliness of all groups of four randomly chosen sequences (*e.g.,* quartets) in a single graph. For each quartet, the three possible unrooted tree topologies are weighted and the posterior weights are plotted using triangular coordinates and mapped onto a triangle. The resulting distribution of points shows whether the data are suitable for a phylogenetic reconstruction or not. Hence, dots localized close to the triangle vertices represent tree-like phylogenetic signal, whereas those close to the center and on the sides represent star-like (completely unresolved) and network-like (partially unresolved) signal, respectively [33]. To facilitate the interpretation of the results, the triangle was partitioned in seven different regions (basins of attraction). Three regions correspond to fully resolved topologies (*A_1_ A_2_* and *A_3_*), one region represents star-like evolution (A_*_) and the three last regions (*A_12_*, *A_13_*, and *A_23_*) reflect the situation where it is difficult to distinguish between two of the three trees (Fig. S1A). The occupancies of each of these regions were then presented in percent. Second, we tested for recombination. Recombination creates mosaic genomes, which violate the assumption of tree-like evolution. Therefore, a network was inferred for each data set using SplitsTree [34]. Each data set was then analyzed for the presence of recombinant sequences using the PHI test [35] with alpha  = 0.001 [36].

## Results

### Assembly

#### De novo assembly and mapping procedure

We used two methods to assemble our eight genomes. First we mapped the reads onto N315. This approach permitted to determine the elements that are in common between N315 and our isolates but gave no indication about the nature of elements present exclusively in our isolates and no idea about possible genome rearrangements. To overcome these two points, we developed a mix *de novo*/mapping procedure. We compared the results obtained with the two methods and observed that our mix *de novo*/mapping procedure gave similar results as the single mapping procedure but permitted to characterize the plasmids and identify the presence of a phage elements. For this reason, we presented and used the results of the mix *de novo*/mapping procedure.

After quality control of the reads and *de novo* assembly of the reads, the contigs obtained were mapped to the reference genome N315. The mapping procedure gave rise to a first alignment of our eight isolates (I1_2001_ to I8_2008_) with N315. This alignment enabled to identify the elements that are common between our isolates and the strain N315 as well as the elements that were absent in our isolates relative to N315. From this alignment, we observed two major differences between ST228 and N315. The first variation was observed in the region corresponding to the *SCCmec* element, the genetic island encoding for the resistance to methicillin. The strain N315 harbored the *SCCmec* type II while our eight isolates had the complete sets of reads corresponding to the SCC*mec* type I. The second difference concerned the region comprising the bacteriophage Φ N315 followed by the pathogenicity island SaPIn1/SaPlm1. Important gaps were observed between the positions 2’007’561 and 2’072’272 of the strain N315 suggesting that these two elements were missing in our eight isolates. Other minor divergences such as mutations, indels and the insertion of a transposase of approximately 1’500 bp in a non coding region of the genome were observed between our isolates and the strain N315. When the two major differences described above and the indels were not considered (by masking them), between 734 and 783 SNPs differentiated our isolates from the genome of the strain N315. Among these, 720 SNPs were common to the eight isolates.

By removing N315, we obtained a second alignment based exclusively on the eight isolates. The total length of this alignment was 2’760’547 bp. The comparison of these 2’760’547 bp revealed that our eight isolates were differentiated by a low number of single mutations plus some differences affecting more than three base pairs (Table 2). Most of these differences consisted of insertions or deletions (indels) of several base pairs (between five and 189 bp). In addition, differences (SNPs and indels) were observed over a sequence of 1200 bp of the isolate I3_2006_. These differences were located in one uncharacterized gene with repeated elements. Without taking into account the differences of Table 2, we observed a total of 163 SNPs (single indels were not considered) with 13 to 90 SNPs differentiating pairs of isolates. Five to 61 private SNPs (*i.e.,* SNPs that were exclusively found in one isolate) were observed for the different isolates.

#### De novo assembly of reads unmapped to N315

In addition to the main alignment representing most of the genome, we found a few elements that did not belong to the chromosome or that could not be positioned on the chromosome. These elements corresponded to reads that did not map to the genome of N315. To determine the constitution of these elements, the non-mapped reads were assembled *de novo*. For each isolate, between 16–17 contigs larger than 1’000 bp were obtained. The annotation and analysis of these contigs indicated that one corresponded to a plasmid and that the others corresponded to phage elements. Interestingly, the presence/absence of these elements varied among isolates. The differences among our eight isolates are described below and illustrated in Figure 2.

The plasmid sequences recovered in our isolates were clearly different from the plasmid sequence of strain N315 (accession number AP003139). The comparison of our plasmid sequences with published database revealed that all our isolates except I5_2006_ had a plasmid closely related to the published plasmid SAP064A (accession number GQ900419). Only two deletions differentiated the plasmids of I1_2001_, I2_2001_ and SAP064A and an additional SNP was observed for the I4_2006_ plasmid. The four other SAP064A-like plasmids exhibited additional minor changes plus the insertion or loss of larger DNA fragments. The plasmids of isolates I3_2006_ and I6_2008_ were characterized by the insertion of a transposase of 1’330 bp and 1’900 bp respectively and the plasmid of isolates I7_2008_ and I8_2008_ by the loss of the whole mercuric operon (*c.a.* 7’000 bp). Finally, we found a completely different plasmid for isolate I5_2006_. This plasmid was closely related to one of the three plasmids found in the USA300 community-acquired MRSA (pUSA03; accession number CP000258; [37]). Our plasmid and pUSA03 differed by only 13 deletions of single base pairs plus 14 SNPs and one 162 bp insertion. The complete sequences of each plasmid were deposited at ENA under the accession numbers given in Table 1.

The remaining 16 contigs obtained after *de novo* assembly represented about 80’000 bp and were constituted of phage elements not present in the strain N315. Interestingly, comparing these contigs with published databases [38], [39] highlighted that they were all constituted of elements found in other published phages but none corresponded exactly to previously described phages. Fifteen out of the 16 contigs were present in all isolates and represented altogether approximately 62’500 bp. The last and largest contig (18’700 bp) was absent from I3_2006_. For technical reasons, it was not possible to determine the complete and exact constitution of these phages and to find their position within the genome. In addition, it was difficult to precisely compare the phage elements among isolates. Although similar, the contigs constituting these phages differed slightly in their size. This is likely explained by the presence of phage elements in unassembled reads or contigs not considered for analyses (*i.e.*, <1000 bp). These unassembled reads and contigs represented *c.a.* 4’000–8’000 bp depending of the isolates (*i.e.*, less than 0.03% of the genome).

### Phylogeny

The phylogenetic tree including N315 clearly confirmed the close relationship among our eight isolates (Fig. 2A). Unfortunately, the resolution of this tree was not sufficient to assess the real link between the eight isolates since bootstrap values were low (Fig. 2B). The tree based exclusively on the eight isolates (Fig. 2C) had a similar topology with branches slightly better supported. Interestingly, mapping the MGEs and the major differences onto this tree (Fig 2D) indicated that all the differences shared between isolates were not monophyletic. This is particularly well exemplified by isolates I7_2008_ and I8_2008_ which did not cluster together on the tree although they had a large number of deletions in common. However, bootstrap supports remained low and it is unclear if these trees reflect the real evolutionary history of the lineage. Detailed analyses of the content of the sequences indicated that there is a low number of informative SNPs; only 41 informative SNPs were identified in the alignment comprising the strain N315 and 44 in the second alignment of the eight isoaltes. Nevertheless, the likelihood mapping indicated high phylogenetic signal (>90%) for the two data sets (Fig. S1B and S1C). In addition, the split decomposition (Fig. S2) and the PHI test found statistically significant evidence for recombination (*P*>0.01 for both data sets).

## Discussion

The aim of the present study was to investigate the short-term evolution of a European clone (ST228) that spread over a ten-year period in a tertiary care hospital. To determine the genetic changes that characterized this lineage, we compared the whole genome of eight isolates collected in the hospital between 2001 and 2008.

The comparison of the eight genomes indicated that these isolates are genetically very closely related (Fig. 2A). Most variations among their genomes consist of SNPs plus a few indels of one to several base pairs (Table 2). In addition to these small variations, more important genetic changes such as the replacement of a plasmid, the loss of phage- or plasmid- elements or the insertion of transposase (Fig. 2D) shaped the genetic diversity of the ST228 lineage that spread within our hospital. Similar acquisitions/losses of mobile genetic elements (MGEs) (*e.g.*, SCC*mec* or bacteriophages) have also been observed in other lineages such as ST5 or ST225 [40], [41]. These and our results indicate that the transfers of MGEs occur locally and probably much more often than previously thought. The impact of such events on the fitness, virulence or epidemicity of the strain is difficult to evaluate. However, it is highly likely that two strains sharing the same genetic background but harboring different plasmids or phages will exhibit differences in their phenotypes.

Interestingly, the survey of MRSA at the tertiary care hospital of Lausanne revealed an important dynamic of clone ST228 over time with a few variants that became dominant while others occurred only sporadically (Fig 1, [7]). Yet, no link between the genetic variations described above and the spreading success of the different variants belonging to ST228 could be established even though the entire genome was scanned. In 2001, 2003 and 2008, outbreaks associated with the clone ST228 took place at the hospital but we found no MGEs (Fig. 2D) or mutations specific of the isolates (I1_2001_, I2_2001_, I7_2008_ and I8_2008_) responsible for these outbreaks. This suggests that the sudden success of some variants is probably not explained by the acquisition or loss of a particular genetic element that is transmitted or inherited from one variant to the others. These results, however, do not mean that the variation in spreading success of the ST228 variants has no genetic basis. First, the genetic factors explaining the spreading success of MRSA might differ from one variant to another. If so, the identification of these factors is especially challenging. Searching for a genetic element common to several strains (*e.g.*, epidemic strains) reduces considerably the number of candidate genes or elements. This is well illustrated by the recent discovery in most strains of the community acquired MRSA USA300 clone of the arginine catabolic mobile element (ACME), which is suspected to favor survival and spreading capacities [37]. In contrast, if epidemicity factors are strain specific, the number of genetic elements that need to be considered is much higher, therefore reducing considerably their possible identification. Second, variations in success of different isolates may also be explained by the acquisition of deleterious genetic changes reducing the spreading capacity of strains originally successful. This hypothesis is supported by the European-wide distribution of the clone ST228 [42] suggesting it has intrinsically a high spreading capacity. It is probable that the genetic changes that have accumulated over time imply more often a burden for the ST228 strains than an improvement of their fitness. Finally, the spreading success of different variants might also be explained by differences in environmental conditions rather than by intrinsic genetic capacities. The hospital setting is complex and multiple parameters such as the use of antimicrobial, the comorbities of the patients, infection control measures (reviewed in [43]), bed occupancy [44] or the number of nurses per patients [44] also affect the dynamic of a clone. We did not notice such changes in our hospital during the last decade. However, variations occurring over very short periods of time or localized in small areas of the hospital in association with stochastic events might create conditions that favor the spread of a strain.

Our last aim was to determine the potential of next generation technologies for very local epidemiology. These technologies are expected to answer fundamental questions about local evolution of pathogens and provide new tools to trace the spread of an isolate both locally and internationally [45]–[49]. The typing methods currently used for studying MRSA (reviewed in [45], [48], [50]) tend to lack the discrimination required for studies performed at local scales. Our study showed that whole genome sequencing provides much more information about the differences characterizing our eight isolates (Table 2, Fig. 2D) than common typing methods; but it is not clear if this additional information is really useful to answer epidemiological issues at very local scales.

Although the likelihood mapping analyses perceived a phylogenetic signal in our data, the low number of SNPs observed in our study and the probable presence of recombination did not permit to obtain reliable information about the real relationship among isolates. Most branches of the trees were not well supported and the tree topology varied depending the part of the genome included in the analyses (core genome based on the differences with N315 *vs* based on the eight isolates, Fig. 2B and 2C respectively) or the algorithm chosen for the alignment (data not shown). Additionally, the fact that the isolates I7_2008_ and I8_2008_ did not cluster together on the trees although they share several significant differences (Fig. 2D) also raised some doubts about the phylogenetic value of these trees. So far, most studies investigating the evolution of pathogens analyzed isolates recovered from the global range of distribution of the clone or species of interest [16], [17], [40], [41]. These studies highlighted geographical signatures in the relationships among isolates. However, this pattern is most likely detectable because the strains that are transferred over large geographical distances inevitably go through bottlenecks followed by rapid expansion enhancing genetic differences among geographical areas [41]. Such signature has not been detected in our study probably because we investigated the evolution of a clone within a single hospital. This local evolution is also suggested by the close relationship among our eight isolates compared to N315 (Fig. 2A). Nevertheless, to confirm the local evolution of our isolates it is crucial to compare the genomes of our isolates with the genomes of other ST228 isolates collected in different hospitals. Such analyses would allow to *i)* establish the global genetic diversity of the lineage, and *ii)* determine the within *versus* between hospitals genetic variations. This information is essential to establish the ratio between local evolution and transfers from other regions. Consequently, as illustrated by the study of clone ST239 [16], it is important to know the genetic background of a lineage at different evolutionary levels for a reliable interpretation of whole genome sequencing data in epidemiological tracking. Thus, understanding the fine scale epidemiology of a particular clone with new technologies requires important investments that need to be adapted for every genetic lineage. In this context, the multiplication of large scale sequencing projects will probably provide important information about many such lineages and will facilitate the use of whole genome sequencing in local epidemiological investigations.

### Conclusion

The sequencing and the genome comparison of eight isolates belonging to a lineage that spread over ten years within a tertiary care hospital revealed that these genomes were extremely closely related. The local evolution of this lineage is mainly driven by point mutations accompanied by few MGE transfers. The rapid evolution of MRSA lineages and their potential phenotypic variations are probably more explained by these MGEs transfers rather than single mutations. Nevertheless, the sudden occurrence of several outbreaks of lineage ST228 at the Lausanne tertiary care hospital did not seem to be explained by a single particular genetic element. Finally, the present study showed that new sequencing technologies improve considerably the quality and quantity of information obtained for a single strain; but this information is still difficult to interpret and needs to be placed into a broader context. Important investments are still necessary for this technology to become useful in routine investigations.

## Supporting Information

Figure S1 **Likelihood Mapping Analysis for the core genomes with and without the strain N315.** Phylogenetic noise was calculated using likelihood mapping analysis as implemented in TREE-PUZZLE [32]. A. Partitioning of the triangle in the seven basins of attraction (see [33] for details). Three basins correspond to fully resolved topologies (*A_1_ A_2_* and *A_3_*), one represents star-like evolution (A_*_) and the three last basins (*A_12_*, *A_13_*, and *A_23_*) reflect the situation where it is difficult to distinguish between two of the three trees. The occupancies (in percent) of the seven basins of attraction for the core genome including the reference strain N315 (B) and for the core genome based exclusively on the eight isolates of ST228 (C).
(DOCX)

Figure S2 **Neighbor-nets for the core genomes with and without the strain N315.** Neighbor-nets for the core genomes with (A) and without (B) the strain N315 were inferred using uncorrected *p*-distances. *P*-values are shown for the PHI test for recombination, where the alpha value is *p* = 0.001.
(DOCX)
