# Supplementary material for: Short Term Evolution of a Highly Transmissible Methicillin-Resistant Staphylococcus aureus Clone (ST228) in a Tertiary Care Hospital
Source: PLoS One. 2012 Jun 18;7(6):e38969. doi: 10.1371/journal.pone.0038969 (PMC3377700; doi:10.1371/journal.pone.0038969)

**Figure S1. Likelihood Mapping Analysis for the core genomes with and without the strain N315**. Phylogenetic noise was calculated using likelihood mapping analysis as implemented in TREE-PUZZLE [32]. A. Partitioning of the triangle in the seven basins of attraction (see [33] for details). Three basins correspond to fully resolved topologies (*A_1_* *A_2_* and *A_3_*), one represents star-like evolution (*A_*_*) and the three last basins (*A_12_*, *A_13_*, and *A_23_*) reflect the situation where it is difficult to distinguish between two of the three trees. The occupancies (in percent) of the seven basins of attraction for the core genome including the reference strain N315 (B) and for the core genome based exclusively on the eight isolates of ST228 (C).


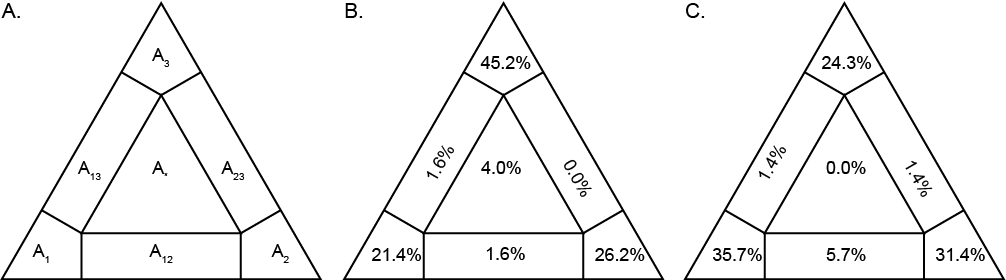

Supplement: Figure S1 — Likelihood Mapping Analysis for the core genomes with and without the strain N315. Phylogenetic noise was calculated using likelihood mapping analysis as implemented in TREE-PUZZLE [32]. A. Partitioning of the triangle in the seven basins of attraction (see [33] for details). Three basins correspond to fully resolved topologies (A1 A2 and A3), one represents star-like evolution (A*) and the three last basins (A12, A13, and A23) reflect the situation where it is difficult to distinguish between two of the three trees. The occupancies (in percent) of the seven basins of attraction for the core genome including the reference strain N315 (B) and for the core genome based exclusively on the eight isolates of ST228 (C). (DOCX) [file pone.0038969.s001.docx]
