# Supplementary material for: Short Term Evolution of a Highly Transmissible Methicillin-Resistant Staphylococcus aureus Clone (ST228) in a Tertiary Care Hospital
Source: PLoS One. 2012 Jun 18;7(6):e38969. doi: 10.1371/journal.pone.0038969 (PMC3377700; doi:10.1371/journal.pone.0038969)

**Figure S2. Neighbor-nets for the core genomes with and without the strain N315.** Neighbor-nets for the core genomes with (A) and without (B) the strain N315 were inferred using uncorrected *p*-distances. *P*-values are shown for the PHI test for recombination, where the alpha value is *p* = 0.001.


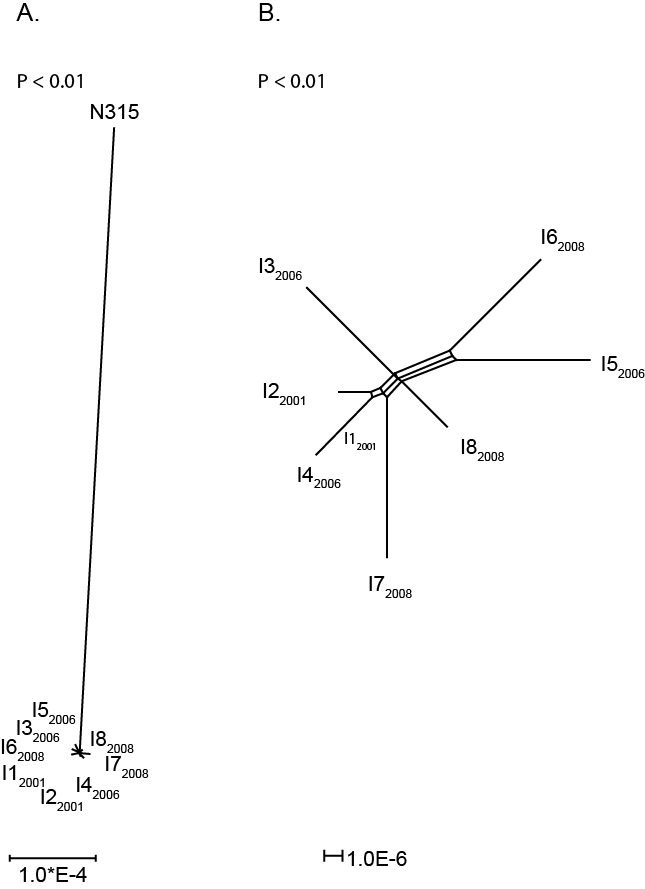

Supplement: Figure S2 — Neighbor-nets for the core genomes with and without the strain N315. Neighbor-nets for the core genomes with (A) and without (B) the strain N315 were inferred using uncorrected p-distances. P-values are shown for the PHI test for recombination, where the alpha value is p = 0.001. (DOCX) [file pone.0038969.s002.docx]
